# Serum secreted frizzled-related protein 5 in relation to insulin sensitivity and its regulation by insulin and free fatty acids

**DOI:** 10.1007/s12020-021-02793-z

**Published:** 2021-06-28

**Authors:** Marta Rydzewska, Agnieszka Nikołajuk, Natalia Matulewicz, Magdalena Stefanowicz, Monika Karczewska-Kupczewska

**Affiliations:** 1 Department of Internal Medicine and Metabolic Diseases, Medical University of Białystok, Białystok, Poland; 2 Department of Prophylaxis of Metabolic Diseases, Institute of Animal Reproduction and Food Research, Polish Academy of Sciences, Olsztyn, Poland; 3 Department of Metabolic Diseases, Medical University of Białystok, Białystok, Poland

**Keywords:** SFRP5, Insulin sensitivity, Free fatty acids, Adiponectin, Hyperinsulinemic-euglycemic clamp

## Abstract

**Purpose:**

Secreted frizzled-related protein 5 (SFRP5) is an adipokine, which acts as an inhibitor of noncanonical WNT signaling pathway. It has been suggested to exert anti-inflammatory and insulin-sensitizing effects, however, contradictory data has also been reported. The aim of this study was to assess serum SFRP5 concentration in a young healthy population in relation to insulin sensitivity and its regulation by hyperinsulinemia and/or serum free fatty acids (FFA) elevation.

**Methods:**

We examined 150 healthy subjects (83 normal-weight and 67 overweight/obese). Insulin sensitivity (M) was measured with hyperinsulinemic-euglycemic clamp. In 20 male subjects, clamp was prolonged to 6 h and after 1 week another clamp with the concurrent Intralipid/heparin infusion was performed. Independent group of 10 male subjects received infusions of Intralipid/heparin or saline in 1-week interval.

**Results:**

Baseline SFRP5 was lower in the overweight/obese group (*p* = 0.01) and was positively associated with M (*r* = 0.23, *p* = 0.006) and serum adiponectin (*r* = 0.55, *p* < 0.001) and negatively with BMI (*r* = −0.18, *p* = 0.03). In multiple regression analysis, adiponectin was independently associated with SFRP5. Insulin infusion resulted in a decrease in serum SFRP5, both at 120′ (*p* = 0.02) and 360′ (*p* = 0.031). This effect was not observed during the clamp with Intralipid/heparin as well as during Intralipid/heparin alone or saline infusions.

**Conclusions:**

The relation between SFRP5 and insulin sensitivity is mainly dependent on adiponectin. FFA abolish a decrease in circulating SFRP5 caused by insulin, but Intralipid/heparin infusion alone does not regulate SFRP5 concentration. Insulin seems to be more important factor in the regulation of circulating SFRP5 levels than FFA.

## Introduction

The worldwide epidemic of overweight and obesity is expanding and has become an alarming problem as well as major health challenge [1]. Obesity-associated disturbances in white adipose tissue (WAT) are linked to the development of insulin resistance and are connected with a chronic state of systemic low-grade inflammation. Furthermore, obesity-related insulin resistance is a major risk factor for type 2 diabetes (T2D), atherosclerosis, and cardiovascular disease [2].

The Wingless-type mouse mammary tumor virus integration site (WNT) family of secreted glycoproteins has established roles in cell differentiation and function in several tissues including WAT [3]. Secreted frizzled-related protein 5 (SFRP5), an anti-inflammatory and insulin-sensitizing adipokine belongs to the SFRPs group, the largest family of WNT inhibitors [4]. SFRP5 is expressed in insulin target tissues such as subcutaneous and visceral adipose tissue, liver, skeletal muscle as well as in beta cells and has been associated with glucose metabolism, obesity, and T2D [5–7]. The antidiabetic effect of SFRP5 is mainly realized by binding with WNT5A to inhibit the activation of c-Jun N terminal kinase (JNK) in noncanonical WNT pathway to reduce secretion of inflammatory factors [6]. In humans, impact of SFRP5 on insulin sensitivity is less known and more controversial. One study showed that in human adipocytes, SFRP5 impairs insulin sensitivity [8], whereas associations between blood concentration of SFRP5 and homoeostasis model assessment of insulin resistance (HOMA-IR) vary between studies [4, 9]. Moreover, the influence of obesity on circulating SFRP5 remains unclear too. It has been reported that no differences existed between lean and obese subjects [9, 10]. On the other hand, some studies showed that circulating SFRP5 concentrations were elevated or decreased in obese and T2D patients [11, 12]. These contradictions implied that the functions of SFRP5 in the pathogenesis of T2D and obesity still are little known.

Adipogenesis is related to glucose and lipid metabolism and plays a role in insulin resistance. This process involves the sequential activation of a cascade of transcription factors that coordinate the expression of genes responsible for the adipogenic phenotype. Peroxisome proliferator-activated receptor γ (PPARγ) and CCAAT/enhancer-binding protein beta (C/EBPβ) are critical regulating factors in early adipocyte differentiation, while adiponectin, inter alia, is responsible for the formation of mature adipocytes [13]. One of the most important regulators of adipogenesis is the canonical WNT/β-catenin signaling pathway. Previous studies have shown that the WNT signaling pathway can inhibit the adipogenesis by suppressing C/EBPβ and PPARγ functions [14]. Genetic evidence demonstrated that noncanonical signaling through WNT5A can antagonize the canonical pathway, but these studies were not focused on adipose tissue metabolism [15]. However, several in vitro studies have previously examined the role of WNT5A in adipogenesis with controversial results [16–18]. Another research demonstrated that SFRP5 was not expressed in preadipocytes, but its mRNA and protein expression levels gradually increased during the differentiation and maturation of adipocytes [19]. However, the interrelationships between SFRP5, insulin sensitivity, and markers of mature adipocytes remain unclear.

Data on the role of SFRP5 in insulin resistance stem mainly from experimental studies and are controversial. Human studies were carried out mostly in people with severe metabolic abnormalities (i.e. T2D, nonalcoholic fatty liver disease) and there is scarce data based on dynamic methods measuring insulin sensitivity. It is relevant to study young, healthy subjects without overt metabolic disturbances to avoid the influence of possible confounding factors. Therefore, the aim of this study was to assess serum SFRP5 concentration in a young healthy population in relation to insulin sensitivity and obesity and its regulation by hyperinsulinemia and/or serum free fatty acids (FFA) elevation to evaluate the link between SFRP5 and insulin sensitivity.

## Materials and methods

### Study participants

The study group consisted of 150 young, healthy volunteers: 117 male subjects and 33 female subjects, 60 males and 23 females of whom were of normal weight (BMI < 25 kg/m2), 57 males and 10 females were overweight/obese [20]. In an additional experiment, designed for Intralipid or saline infusions, we examined 10 male subjects with similar characteristics described previously [21]. All study participants were nonsmokers, without serious disease or morbid obesity, and not taking any drugs. Subjects were excluded if they had any inflammatory disease within the last 3 months. All subjects had no clinical and laboratory signs of inflammation and had not taken anti-inflammatory drugs within the last 3 months. Anthropometric measurements were performed as previously described [22]. Participants underwent clinical examination and appropriate laboratory tests. Body weight of the subjects had remained stable for at least 3 months before the study. The study protocol was approved by the local ethics committee of the Medical University of Białystok. A written informed consent was obtained from all volunteers before their participation in the study.

### Glucose tolerance and insulin sensitivity

A standard oral glucose tolerance test (OGTT) was performed and all subjects had normal glucose tolerance according to World Health Organization criteria. After 1 week, insulin sensitivity (M) was assessed with the 2-h hyperinsulinemic-euglycemic clamp (HEC) technique, as previously described [23] and was calculated per fat-free mass (ffm). In the subgroup of 20 subjects, 9 normal-weight and 11 overweight/obese the clamp was prolonged to 6 h. After one week, another 6-hour clamp, with concurrent Intralipid/heparin infusion, was performed as described [22]. No difference in steady-state insulin concentration between these protocols was observed.

In an independent group of 10 male subjects, 4 normal-weight and 6 overweight, 4-h Intralipid/heparin and saline infusions were performed in 1-week interval as previously described [21].

Since the experiments were performed in 1-week interval, only males were studied to exclude the potential influence of menstrual cycle.

### Biochemical procedures

Plasma glucose, serum lipids, and insulin were measured as previously described [21, 23]. Serum adiponectin was measured with a radioimmunoassay kit (Millipore, St. Charles, Missouri, USA) with the detection limit of 1 ng/ml and with intra-assay and inter-assay coefficients of variation (CVs) below 6.3 and 9.5%, respectively. Serum SFRP5 was measured with ELISA Kit (Cusabio Biotech Co., Ltd., Wuhan, China) with the detection limit of 0.39 pg/ml and with intra-assay and inter assay CVs below 8 and 10%, respectively.

### Statistical analysis

Statistics were performed with the STATISTICA 12.5 program (StatSoft, Krakow, Poland). All data are presented as mean ± standard deviation. Variables, which did not have a normal distribution, were log transformed before analyses. For data presentation, these variables were antilog transformed again to absolute values. Differences between normal-weight and overweight/obese individuals were analyzed with unpaired Student’s *t* test. To assess the effects of sex and age, we used analysis of covariance. Differences in serum SFRP5 concentrations during both clamps and Intralipid/heparin or saline infusions were assessed with repeated-measures ANOVA with post-hoc Tukey test. The relationships between variables were studied with the Pearson product moment correlation analysis and with multiple regression analysis. The level of significance was accepted as *p* < 0.05.

## Results

### Serum SFRP5 concentrations and its association with clinical and biochemical parameters

**Fig. 1:**
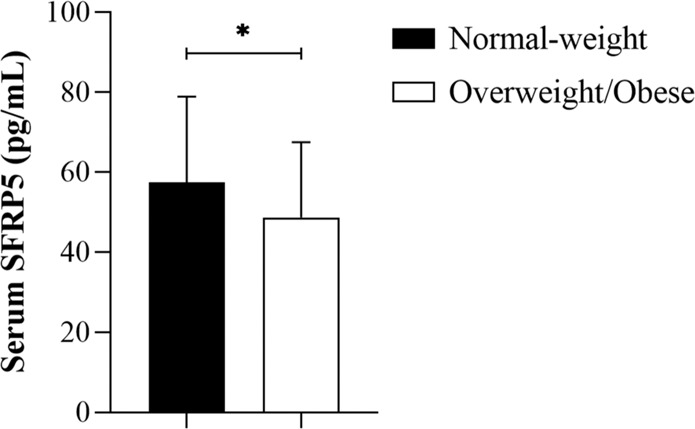
Circulating SFRP5 levels according to BMI (normal-weight: BMI < 25 kg/m^2^ and overweight/obese: BMI ≥ 25 kg/m^2^). Values are given as means ± SD, *p < 0.05

Baseline characteristics of the study participants divided into normal-weight and overweight/obese subgroups and the results of blood tests are presented in Table 1. Insulin sensitivity was lower in the overweight/obese group in comparison with the normal-weight group (*p* = 0.037). Importantly, fasting SFRP5 levels were significantly lower in overweight/obese subjects in comparison with normal-weight subjects (*p* = 0.01, Fig. 1). This difference was still significant after adjustment for age and sex.

**Table 1:** Clinical and biochemical characteristics of the study groups

	Normal- weight (*n* = 83)	Overweight/obese (*n* = 67)
Age (years)	22.73 ± 2.08	24.22 ± 3.56*
BMI (kg/m^2^)	22.31 ± 1.68	28.63 ± 3.36*
Waist circumference (cm)	80.42 ± 5.57	90.51 ± 10.87*
% body fat	17.78 ± 6.79	27.61 ± 8.27*
Fasting plasma glucose (mg/dL)	80.12 ± 7.82	87.62 ± 8.90
Plasma glucose at 120 min OGTT (mg/dL)	81.99 ± 20.09	83.61 ± 17.87
Fasting serum insulin (μIU/mL)	9.40 ± 4.41	13.58 ± 6.64*
M (mg/kg ffm/min)	7.63 ± 2.88	6.63 ± 3.00*
Cholesterol (mg/dL)	163.13 ± 29.33	179.32 ± 31.00*
Triglycerides (mg/dL)	75.46 ± 29.98	102.93 ± 54.59*
HDL-cholesterol (mg/dL)	62.68 ± 11.97	55.85 ± 10.68*
LDL-cholesterol (mg/dL)	93.33 ± 32.07	108.97 ± 31.41*
Adiponectin (µg/mL)	17.35 ± 6.80	14.60 ± 6.74*

*M* insulin sensitivity.

*p < 0.05.

We next investigated the relationship of circulating SFRP5 levels with various clinical and biochemical parameters by using partial correlations (Table 2). Serum SFRP5 positively associated with insulin sensitivity (*r* = 0.23, *p* = 0.006), HDL-cholesterol (*r* = 0.25, *p* = 0.003) and negatively with BMI (*r* = −0.18, *p* = 0.03) waist circumference (*r* = −0.2, *p* = 0.16), triglycerides (*r* = −0.25, *p* = 0.002), and fasting serum insulin (*r* = −0.27, *p* = 0.001). Interestingly, we also observed strong positive correlations of serum SFRP5 with circulating concentrations of adiponectin (*r* = 0.55, *p* < 0.001) and this association remained highly significant in multiple regression analysis (*β* = 0.50, *p* < 0.0001), whereas association between SFRP5 and insulin sensitivity was no longer significant after adjusting for serum adiponectin.

**Table 2:** Correlations between circulating SFRP5 and other estimated parameters

SFRP5	Entire study group (*n* = 150)	
	*r*	*p*
BMI	−0.17	0.03
Waist circumference	−0.20	0.02
Fasting serum insulin	−0.27	0.001
M	0.23	0.006
Triglycerides	−0.25	0.002
HDL-cholesterol	0.25	0.003
Adiponectin	0.55	0.0001

Correlation coefficients (Pearson’s *r*) are shown

### The effects of hyperinsulinemia with/no Intralipid/heparin infusion on circulating SFRP5 levels

**Fig. 2:**
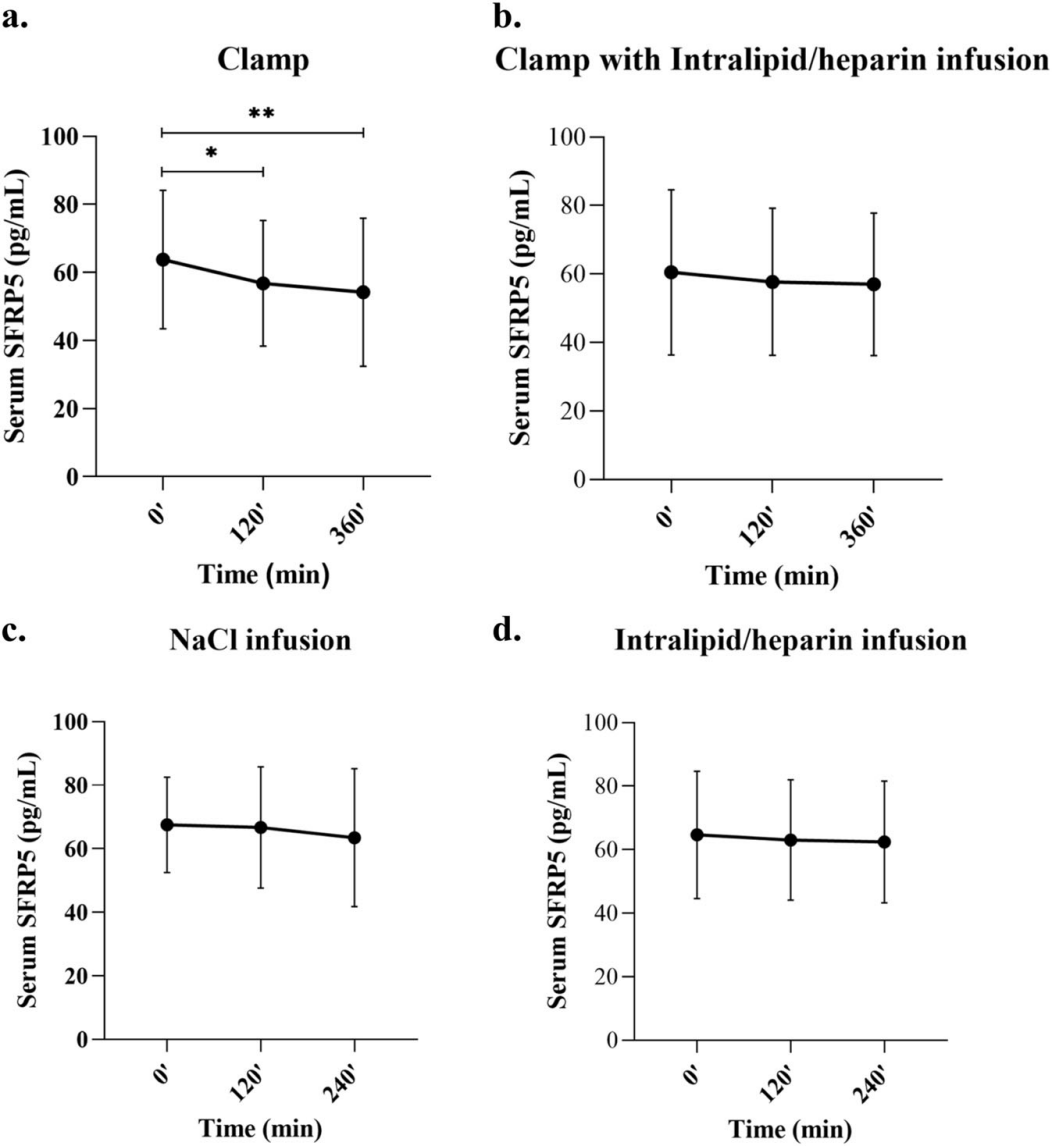
Effects of hyperinsulinemia, hyperinsulinemia with Intralipid/heparin infusion, Intralipid/heparin infusion, and saline infusion on circulating SFRP5 concentrations. Serum SFRP5 levels in healthy subjects during: **a** HEC (vs 0 min, *p < 0.05, **p < 0.001), **b** HEC with Intralipid/heparin infusion, **c** saline infusion, **d** Intralipid/heparin infusion. Values are given as means ± SD

In response to hyperinsulinemia during 6-h HEC, circulating SFRP5 in 20 healthy subjects significantly dropped from 63.78 ± 20.35 pg/mL to 56.8 ± 18.46 pg/mL at 120 min (*p* = 0.02), then to 54.19 ± 21.73 pg/mL at 360 min (*p* = 0.001) (Fig. 2a). The effect of insulin on serum SFRP5 was similar in normal-weight individuals (0′, 69.26 ± 23.11 pg/mL; 120′, 61.91 ± 18.56 pg/mL; 360′, 60.32 ± 23.68 pg/mL; both *p* < 0.05 vs 0′)and in overweight/obese individuals (0′, 57.62 ± 15.94 pg/mL; 120′, 51.04 ± 17.71 pg/mL; 360′, 47.30 ± 18.33 pg/mL; both *p* < 0.05 vs 0′). This effect was not observed during the 6-h HEC with Intralipid/heparin infusion, as SFRP5 was stable throughout this study (Fig. 2b), both in normal-weight (0′ 73.98 ± 22.40 pg/mL; 120′, 70.92 ± 20.06 pg/mL; 360′, 70.14 ± 17.80 pg/mL) and in overweight obese individuals (0′ 48.42 ± 19.36 pg/mL; 120′, 45.87 ± 15.19 pg/mL; 360′, 45.20 ± 16.28 pg/mL).

### The effects of Intralipid/heparin or saline infusion on circulating SFRP5 levels

The infusion of saline, in another group of 10 healthy subjects, had no effect on circulating concentrations of SFRP5 (Fig. 2c), both in normal-weight individuals (0′, 71.85 ± 18.49 pg/ml; 120′, 72.84 ± 16.90 pg/mL; 240′, 68.01 ± 15.45 pg/mL) and in overweight/obese individuals (0′, 64.67 ± 17.33 pg/mL; 120′, 62.66 ± 20.83 pg/mL; 240′, 60.48 ± 20.06 pg/mL). Furthermore, in response to 4-h infusion of Intralipid/heparin alone, circulating SFRP5 remained unchanged throughout the study (Fig. 2d), both in normal-weight individuals (0′, 74.88 ± 16.86 pg/ml; 120′, 72.99 ± 14.39 pg/mL; 240′, 73.48 ± 13.37 pg/mL) and in overweight/obese individuals (0′, 57.86 ± 20.31 pg/mL; 120′, 56.43 ± 19.80 pg/mL; 240′, 55.12 ± 19.83 pg/mL).

## Discussion

To our knowledge, this study is the first to report that acute elevation of FFA does not influence the concentrations of SFRP5 in humans and hyperinsulinemia per se rather than lipids leads to a decreased SFRP5 levels in young, healthy subjects without overt metabolic disturbances. Moreover, our findings may suggest that the relationship between SFRP5 and insulin sensitivity is mostly dependent on adiponectin. And last but not least, we described the declined circulating SFRP5 concentrations in the overweight/obese group. Many factors may affect the secretion of SFRP5, such as the extent of obesity, glycemic status, medications, and other diseases and complications. In the present study, subjects were healthy, young with normal glucose tolerance thus these may strengthen the analysis in the current study.

Previous studies on the regulation of SFRP5 in obesity both in in vitro and in vivo showed conflicting data. In our work we demonstrated the reduced concentration of SFRP5 in overweight and moderately obese individuals as well as the negative correlation of baseline SFRP5 concentration with indicators of adiposity. This is in line with other human studies showing that plasma levels of SFRP5 were decreased in obese people and the circulating SFRP5 was associated inversely with BMI, waist circumference, FFA, triglycerides, fasting serum insulin, and positively with HDL-cholesterol [12, 24, 25]. On the other hand, Schulte et al. showed that circulating SFRP5 levels were not influenced by obesity but were increasing with weight loss in obese individuals. However, the study population consisted only of subjects with obesity with mean BMI within the range of morbid obesity. Moreover, this and our study were markedly different regarding the sample size and age of participants [10]. In another study with young Chinese population consisting of 80 obese individuals and 80 nonobese controls there were no significant difference in circulating SFRP5 concentration between groups. Additionally, authors did not observe any significantly associations between SFRP5 concentration and biochemical parameters of metabolic syndrome as well as anthropometric measurements [19]. We can suppose that the reason for this discrepancy with our findings might be a different inclusion criteria and possibly different ethnicity [26]. Most of studies which focused on the role of SFRP5 in humans were performed on Asians. There is a lack of studies conducted on Caucasian cohorts, especially with young subjects.

In our study we measured insulin sensitivity using the gold standard method and our results indicated the positive correlation between circulating SFRP5 and insulin sensitivity which is in line with previous reports suggesting a protective role of SFRP5 as an anti-inflammatory marker in the development of T2D [12, 27, 28]. Additionally, other authors demonstrated during in vitro experiments, up‐regulation of SFRP5 expression in 3T3-L1 adipocytes inhibited the inflammatory, insulin-resistant state by blocking WNT5A activity [29]. Moreover, authors suggest consentaneously that an increase in the ratio of WNT5A to SFRP5 is linked to inhibited glucose and insulin signaling [12, 30]. In details, the animal studies revealed that the activation of JNK-1 by WNT5A impairs the activity of a target protein called insulin receptor substrate-1 (IRS-1) leading to suppressed insulin signaling and development of insulin resistance [30]. In contrast, other cross-sectional studies including people with and/or without T2D found that circulating SFRP5 showed a positive [31] or no association [9] with insulin resistance. However, so far there was only one study, with 30 healthy women, in which insulin sensitivity was measured with HEC and the results were consistent with our study [27]. It is worth mentioning that during HEC normal plasma glucose level is maintained, thus we may exclude the potential effect of hyperglycemia on circulating SFRP5 levels. The method of measurement of insulin sensitivity and the different inclusion criteria in mentioned studies may be of importance.

We also observed a strong positive correlations of plasma SFRP5 with circulating concentrations of adiponectin and in the fully adjusted model higher SFRP5 levels remained independently associated with higher adiponectin levels. Our study is in line with previous reports describing positive correlations between both adipokines [27, 28]. This novel finding may suggest that the relationship between SFRP5 and insulin sensitivity is mainly dependent on adiponectin. Numerous studies have demonstrated that adiponectin has insulin-sensitizing, anti-atherogenic, and anti-inflammatory properties which have similar effects to SFRP5 [32]. Furthermore, a recent study demonstrated that WNT5A expression in WAT is significantly decreased in adiponectin-overexpressing mice [33]. Thus these observations may suggest that suppression of WNT5A is a common mechanism by which both SFRP5 and adiponectin demonstrate their anti-inflammatory and insulin-sensitizing actions.

Furthermore, adiponectin protein synthesis and secretion occur exclusively in mature adipocytes and therefore it is described as a distinctive marker of adipocyte differentiation [34]. Interestingly, recent in vitro studies indicated highly increased concentration of SFRP5 in mature adipocytes rather than preadipocytes and thus these findings may indicate that SFRP5 represents a candidate for a mature adipocyte marker gene [19]. There is evidence that the dysfunction of adipose tissue, rather than the degree of adiposity, may cause metabolic abnormalities including insulin resistance. The insufficiency of adipose tissue may manifest in a lower expression of transcription factors, like PPARγ, and a lower adiponectin secretion [35]. Recent study showed that SFRP5 was a target gene under the direct transcriptional regulation PPARγ in 3T3- L1 adipocytes [36]. At the same time, it is worth mentioning that PPARy regulates adiponectin gene expression, processing, and secretion [37]. Taken together, we can speculate that decreased concentrations of SFRP5 in overweight/obese individuals may be the effect of impaired functions of mature adipocytes and SFRP5 effects are associated with adiponectin. In contrast to evidence on relatively novel adipokine which is SFRP5, it is well documented that adiponectin modulates multiple signaling pathways to exert its physiological and protective functions and therefore further research focused on investigating the possible common pathways between both proteins is needed.

During hyperinsulinemia, we observed a marked decrease in serum SFRP5 concentrations. These results lead us to speculate that short-term hyperinsulinemia may have an inhibitory effect on SFRP5 secretion and/or release. In general, hyperinsulinemia is associated with obesity [38]. As our report shows, during the HEC the action of hyperinsulinemia on serum SFRP5 concentration imitates the differences observed in individuals with overweight/obesity in the baseline state. In line with our results, downregulation of circulating SFRP5 by hyperinsulinemia obtained during HEC in healthy women has been reported in the study by Hu et al. [27].

It is well documented that short-term Intralipid/heparin infusion significantly increases FFA levels and induces insulin resistance by decreasing peripheral glucose uptake and down-regulating intracellular insulin signaling [39]. To investigate the effect of lipid-induced insulin resistance on circulating SFRP5 in vivo, we examined the alterations of serum SFRP5 level during lipid infusion combined with HEC. We observed that SFRP5 concentration was stable throughout the clamp. We hypothesized that in the insulin-resistant condition caused by an acute elevation of serum FFA, the inhibitory effect of insulin on SFRP5 secretion might be abolished. To check this hypothesis, we extended our study and performed infusion of Intralipid/heparin or saline in another subgroup of subjects. In both conditions the concentrations of circulating SFRP5 did not differ and were stable. It should be noted that there were different time-points of final sampling between the clamp with/without Intralipid/heparin (6 h) and between Intralipid/heparin alone and saline infusions (4 h). However, the effect of insulin on serum SFRP5 was already been observed at 120 min the clamp without Intralipid/heparin, whereas it was absent in other experiments at the same time-point. Furthermore, in the clamp without Intralipid/heparin serum SFRP5 was comparable at 120 min and 360 min of the experiment. During the clamp, metabolic conditions remain stable between 4 and 6 h. Therefore, these data indicate that insulin exerts its effect rapidly (2 h are sufficient) and the decrease in serum SFRP5 is maintained for at least 6 h during hyperinsulinemia. Lack of change in serum SFRP5 at 2 and 4 h of the Intralipid/heparin alone and saline infusion allows to assume that the changes observed during the clamp without Intralipid/heparin reflect specific insulin effect.

To the best of our knowledge, there is a lack of evidence of the elevated FFA effects on circulating SFRP5 in humans. The addition of heparin to the Intralipid infusion releases lipoprotein lipase into the bloodstream, thus facilitating the breakdown of circulating triglycerides, therefore heparin is necessary to increase circulating FFA. The protocol we used is an established method to induce insulin resistance. It is well documented that increased FFA inhibit insulin action [39]. Our data indicate that Intralipid/heparin infusion abolishes insulin action in hyperinsulinemic state, however, Intralipid/heparin infusion alone does not have any effect on circulating SFRP5. Therefore, one may assume that the lack of change in serum SFRP5 during the clamp with Intralipid/heparin is associated with the abolishment of insulin action, which is attributable to an increased circulating FFA. To the best of our knowledge, no data are available on the possible effect of heparin on circulating SFRP5. Together, our results suggest that the decreased SFRP5 concentrations are directly associated with insulin effects rather than lipids.

In conclusion, the present study provided novel evidence that hyperinsulinemia decreases serum SFRP5 and that the concurrent elevation of FFA abolishes this effect, but Intralipid/heparin infusion alone does not regulate SFRP5 concentration. Thus, insulin seems to be more important factor in the regulation of circulating SFRP5 levels. Furthermore, we suggest for the first time that the relationship between SFRP5 and insulin sensitivity is mainly dependent on adiponectin.

## Data Availability

Data from the present study are available from the corresponding author upon reasonable request.
